# Tight Junction Protein 1 Suppresses Kidney Renal Clear Cell Carcinoma Cells Proliferation by Inducing Autophagy

**DOI:** 10.7150/ijms.81065

**Published:** 2023-09-11

**Authors:** Miao Li, Di-Sheng Zhou, Xin-Rong Shao, Sze-Hoi Chan, Zhao-Xia Dong, Xue-Qi Liu, Shu-Na Chen, Lin Qi, Luis Velasquez Zarate, Xiao-Mei Wang, Xin Du, Xing-Ding Zhang

**Affiliations:** 1 Shenzhen Key Laboratory for Systems Medicine in Inflammatory Diseases, School of Medicine, Shenzhen Campus of Sun Yat-Sen University, The Seventh Affiliated Hospital of Sun Yat-sen University, Sun Yat-sen University, Shenzhen, China.; 2 Medical College of Georgia-Augusta University, Augusta, GA 30912, United States.; 3 Department of Hematology and Shenzhen Bone Marrow Transplantation Public Service Platform, The First Affiliated Hospital of Shenzhen University, Shenzhen Second People's Hospital, Guangdong Key Laboratory for Biomedical Measurements and Ultrasound Imaging, National-Regional Key Technology Engineering Laboratory for Medical Ultrasound, School of Biomedical Engineering, Shenzhen University Medical School, Shenzhen University, Shenzhen 518060, China.

**Keywords:** KIRC, Tight junction protein 1, autophagy, proliferation

## Abstract

TJP1, an adaptor protein of the adhesive barrier, has been found to exhibit distinct oncogenic or tumor suppressor functions in a cell-type dependent manner. However, the role of TJP1 in kidney renal clear cell carcinoma (KIRC) remains to be explored. The results showed a marked down-regulation of TJP1 in KIRC tissues compared to normal tissues. Low expression of TJP1 was significantly associated with high grade and poor prognosis in KIRC. Autophagosome aggregation and LC3 II conversion demonstrated that TJP1 may induce autophagy signaling in 786-O and OS-RC-2 cells. Knockdown of TJP1 led to a decrease in the expression of autophagy-related genes, such as BECN1, ATG3, and ATG7. Consistently, TJP1 expression showed a significant positive correlation with these autophagy-related genes in KIRC patients. Furthermore, the overall survival analysis of KIRC patients based on the expression of autophagy-related genes revealed that most of these genes were associated with a good prognosis. TJP1 overexpression significantly suppressed cell proliferation and tumor growth in 786-O cells, whereas the addition of an autophagy inhibitor diminished its inhibitory function. Taken together, these results suggest that TJP1 serves as a favorable prognostic marker and induces autophagy to suppress cell proliferation and tumor growth in KIRC.

## Introduction

Renal cell carcinoma (RCC) is a prevalent urological tumor, constituting approximately 2.2% of the new cases among 36 types of adult malignancies worldwide in 2020^1^. It is the third most common urological cancer after prostate and bladder cancer, but it has the highest mortality rate at over 40% with the dramatic increase in incidence during the last decades. Kidney renal clear cell carcinoma (KIRC) is the most common subtype of RCC and accounts for approximately 80% of these tumors^2^. KIRC is characterized by high pathological stages and resistance to chemotherapy and radiotherapy^3^. Patients of KIRC are often diagnosed at the advanced stage with poor prognosis lacks specific diagnostic markers. Therefore, better understanding the pathogenesis of KIRC and identifying new biomarkers for KIRC are urgent research objectives.

Autophagy has been shown to have both tumor suppressive and tumor promoting functions during cancer progression^4^. The tumor suppressive function of autophagy is partly attributed to its ability to induce cell death via several mechanisms. While autophagy sustain energy homeostasis and mitochondrial metabolism, cancer cells could remodel autophagy signaling to meet the metabolic demands of uncontrolled cell proliferation^5^, ^6^. In addition, in various mouse models, activation of P53-mediated suppressive signaling pathway was observed when autophagy was ablated, suggesting that autophagy may serve as a negative regulator of P53 signaling pathway^7^. Autophagy also has an oncogenic role in diverse cancers, such as the activation of RAS oncogenic signaling pathway^8^. Several pharmaceutical agents targeting autophagy in renal cancer have been proven effective. Among them, sunitinib, a multitargeted tyrosine kinase inhibitor, can induce cancer cells autophagy to markedly prolong the overall survival of patients of advanced renal cell carcinoma^9^, ^10^. Conversely, Chloroquine and PI3K inhibitors can effectively inhibit the autophagic flux and promote apoptosis of cancer cells. Therefore, it remains unclear whether we should induce or inhibit autophagy in the treatment of cancer^11^.

Tight junction protein 1 (TJP1), also known as zonula occludens 1 (ZO-1), is the component of tight junctions that form adhesive barriers and plays an important role in signal transduction^12^, ^13^. TJP1 directly interacts with the PDZ domain and the C-terminus of claudins^14^ to form tight junctions. TJP1 binds to G-protein-coupled receptors through its GUK domain to mediate several signaling pathway^15^. Accumulating evidence have suggested that TJP1 plays a key role in cancer development and progression, such as tumor vascular normalization, tumor chemoresistance and effective mucosal repair^16^-^18^. Generally, TJP1 has been reported to function as a tumor suppressor in many earlier studies. TJP1 is downregulated in many cancer types and low expression of TJP1 correlates with advanced stage and poor prognosis^19^. Recently, some studies have reported that TJP1 promotes cancer cell proliferation and cell motility in some cancer types^20^, ^21^, such as bladder cancer, pancreatic cancer, colorectal cancer, melanoma, and non-small cell lung cancer (NSCLC)^22^-^25^. However, the role of TJP1 in KIRC still remains unknown.

In our study, we observed a significant correlation between low TJP1 expression and tumor grade as well as poor prognosis in KIRC. Additionally, our research demonstrated that the overexpression of TJP1 induced autophagy signaling, resulting in the inhibition of cell proliferation and tumor growth in both KIRC cells and xenograft models. Furthermore, we discovered a positive correlation between the expression of autophagy-related genes, TJP1, and a favorable prognosis in KIRC. Collectively, these findings suggest that TJP1 suppresses cell proliferation and tumor growth by inducing autophagy in KIRC cells, which could serve as a promising prognostic indicator in KIRC.

## Materials and methods

### Cell culture

The human KIRC cell line 786-O and OS-RC-2 was cultured in a humidified incubator at 37°C with a controlled atmosphere of ambient air 5% CO_2_. Cells were grown in RPMI-1640 supplemented with 10% FBS. 293T cells were grown in DMEM supplemented with 10% FBS. All cell lines used in this research were authenticated by using shorttandem repeat profiling > 6 months ago when this project was initiated and were cultured no > 2 months.

### PCR, plasmid construction and transfection

The CDS of human TJP1 (NCBI Gene ID: 7082) was amplified by reverse transcription PCR and cloned into the pSin-DOX plasmid vector. shRNA targeting TJP1 was cloned into the pLKO.1-puromycin transfer plasmid (ATCC #8453) as indicated before^18^. The sequences for shRNA are listed below: CCGGGCGATCTCATAAACTTCGTAACTCGAGTTACGAAGTTTATGAGATCGCTTTTTG and CCGGGCCTGCATACAATAAAGCAAACTCGAGTTTGCTTTATTGTATGCAGGCTTTTTG. The nontargeted control shRNA contained the insert TTCTCCGAACGTGTCACGT, which has no homology with any known human gene transcripts.

### Lentivirus package and stable cell line construction

The lentivirus package used in this study contained the pAX2 packaging plasmid and pMD2G envelope plasmid. All recombinant lentiviruses were produced by transient transfection of 293T cells according to standard protocols. Briefly, subconfluent 293T cells were transduced with 20 μg of an expression vector, 15 μg of pAX2 and 5 μg of pMD2G by PEI Max transfection reagent (Polysciences, 24765). After 6 h, the medium was changed, and recombinant lentivirus was harvested twice at 48 h and 72 h later. After centrifugation at 1800 rpm, the supernatants were used to infect 786-O cells in growth medium containing 6 μg/mL polybrene (Beyotime, C0351). Five days after puromycin (InvivoGen, ant-pr-1) selection, pooled cells were evaluated for their gene and protein expression.

### RNA extraction and real-time PCR

Total RNA was extracted using TRIzol (Invitrogen, 15596018), and 1 μg of total RNA product was reverse transcribed by using the Reverse Transcription Kit (Takara, RR047A) to obtain cDNA. Real-time PCR was performed using SYBR Green reagent (Takara TB Green, RR420A), and the mRNA level was detected by an ABI Step-one Detection System. The primers used in the real-time PCR assay were as follows: GAPDH-F: GGAGCGAGATCCCTCCAAAAT; GAPDH-R: GGCTGTTGTCATACTTCTCATGG; TJP1-F: ACCAGTAAGTCGTCCTGATCC; TJP1-R: TCGGCCAAATCTTCTCACTCC; BECN1-F: ACCTCAGCCGAAGACTGAAG; BECN1-R: AACAGCGTTTGTAGTTCTGACA; ATG5-F: AAAGATGTGCTTCGAGATGTGT; ATG5-R: AAAGATGTGCTTCGAGATGTGT; ATG7-F: ATGATCCCTGTAACTTAGCCCA; ATG7-R: CACGGAAGCAAACAACTTCAAC; ULK1-F: AGCACGATTTGGAGGTCGC; ULK1-R: GCCACGATGTTTTCATGTTTCA; ATG16L2-F: TGGACAAGTTCTCAAAGAAGCTG; ATG16L2-R: CCTCAGTGCGACCAGTGAT.

### Protein extraction and Western blot

Cell lysates for protein extraction were collected in RIPA lysis buffer containing protease inhibitor cocktail (Thermo, 78443) for 30 min on ice and centrifuged at 12000 × g for 10 min at 4°C to obtain supernatant. Protein quantification was performed with the Bradford Protein Assay Kit (Thermo, 23236). Protein was separated by SDS-PAGE electrophoresis followed by transfer to PVDF membranes (Millipore, ISEQ00010). After blocking with 5% skim milk (BD Difco, 232100) in Tris-buffered saline containing 0.5% Tween 20 for 1 h at room temperature, the membranes were incubated with the corresponding antibodies overnight at 4°C. The targeted proteins were visualized by an ECL detection kit (NCM Biotech, P10300) after incubation with HRP-conjugated antibodies. The primary antibodies included the following: TJP1 (Abcam, ab216880), HSP90 (Proteintech, 66318), BECN1 (ABclonal, A7353), p62 (ABclonal, A11483), and LC3 (Sigma, L7543).

### Cell proliferation and viability assays

786-O (2 × 10^3^ cells/well) were seeded into a 96-well cell culture plate and incubated for 0 h, 24 h, 48 h and 72 h. Cell viability and proliferation curves were assessed with CCK8 kits (Vazyme, A311-02), and the optical density (OD) at 450 nm was assessed with an EPOCH spectrophotometer (BioTek).

### Immunofluorescence assay

For the immunofluorescence assay, cells were cultured on glass-bottom dishes. 786-O cells were fixed with 4% paraformaldehyde and subjected to membrane permeabilization with 0.2% Triton, blocking with sheep serum for 1 h and antibody incubation overnight. Nuclei were stained with 4,6-diamidino-2-phenylindole (DAPI, Invitrogen, 62248) and captured by a microscope (Eclipse Ti2E, NIKON). The primary antibodies included the following: LC3 (Sigma, L7543).

### Nude mouse xenografts

Female 4-week-old BALB/c nude mice were purchased from the Sun Yat Sen Experimental Animal Centre (Guangzhou, China). All studies were approved and supervised by the Animal Care and Use Committee of Sun Yat Sen Experimental Animal Centre. Exponentially growing 786-O-Dox cells (1×10^6^ in 0.1 ml medium) were inoculated subcutaneously into the back of the mouse. Mice were divided into three groups when the tumor volume reached approximately 20 mm^3^. 3-MA was dissolved in 100 μl PBS and injected intraperitoneally every 3 days until the end of the experiment. The tumor volume was calculated as follows: V(mm^3^) = (length ×width^2^)/2. The mice were sacrificed 50 days after inoculation, and the tumor tissue was subcutaneously dissected to obtain the tumor bearing. The tumor tissue was rinsed in precooled PBS to remove blood, dried with filter paper, cut into pieces with scissors, weighed, placed in a tube, and lysed in RIPA buffer containing protease inhibitors at a ratio of 100 mg/mL liquid. The tube was placed into a tissue sample crusher for shaking at 15 Hz for 10 min and then lysed on ice for 30 min. Then, the protein sample was centrifuged at 12000 × g for 10 min, and the supernatant was aspirated carefully into a new tube and stored at -80°C.

### Statistical analysis

For data analysis, we used GraphPad Prism 8 software (San Diego, 265 California) and performed a two-tailed t test and two-way analysis of variance (ANOVA) to analyze the statistically significant differences. Kaplan-Meier survival analysis was analyzed by log-rank test and Cox regression analysis.

## Results

### Low TJP1 expression correlates with advanced tumor grade and poor prognosis in KIRC

**Figure 1:**
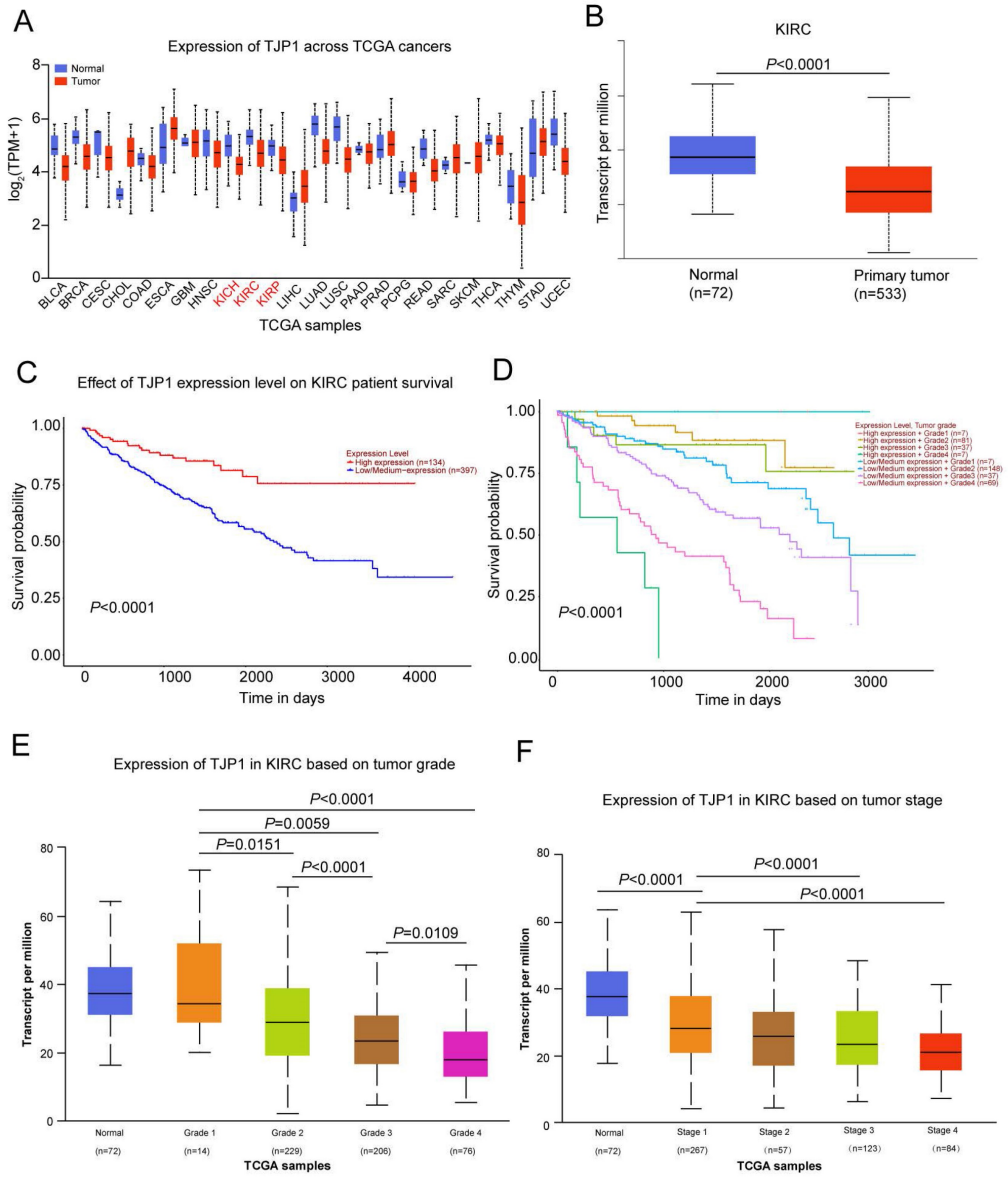
** Downregulation of TJP1 correlates with tumor grade and poor prognosis in KIRC (A)** TJP1 expression in the pan-cancer (TCGA database). **(B)** TJP1 expression in primary KIRC tissues (n=533) and normal tissues (n=72), *P*<0.0001. **(C)** The overall survival rate was analyzed based on TJP1 expression level in KIRC patients. n=531, *P*<0.0001. **(D)** The overall survival rate was analyzed based on combining TJP1 expression with tumor grades in KIRC patients. n=393, *P*<0.0001. **(E)** The expression of TJP1 based on tumor grade in KIRC. **(F)** The expression of TJP1 based on tumor stage in KIRC.

We used the UALCAN website (http://ualcan.path.uab.edu/) to detect TJP1 expression in 24 common tumors from TCGA database, which revealed that TJP1 was downregulated in kidney chromophobe (KICH), kidney renal clear cell carcinoma (KIRC), and kidney renal papillary cell carcinoma (KIRP) compared to normal tissues (Fig. 1A). As KIRC accounts for 80% of all kidney cancers, we next focused on investigating the function and mechanism of TJP1 in KIRC. The TJP1 expression was markedly downregulated in 533 KIRC tissues compared to 72 normal tissues, and low expression of TJP1 positively correlated with poor prognosis in KIRC (Fig. 1B-C). Further analysis revealed that low TJP1 expression also correlated with poor overall survival in KIRC patients with same tumor grade (Fig. 1D). Moreover, low TJP1 expression was significant associated with higher tumor grade and pathologic stage (Fig. 1E-F), indicating TJP1 is a favorable prognostic marker in KIRC.

### TJP1 regulates autophagy in KIRC cells

**Figure 2:**
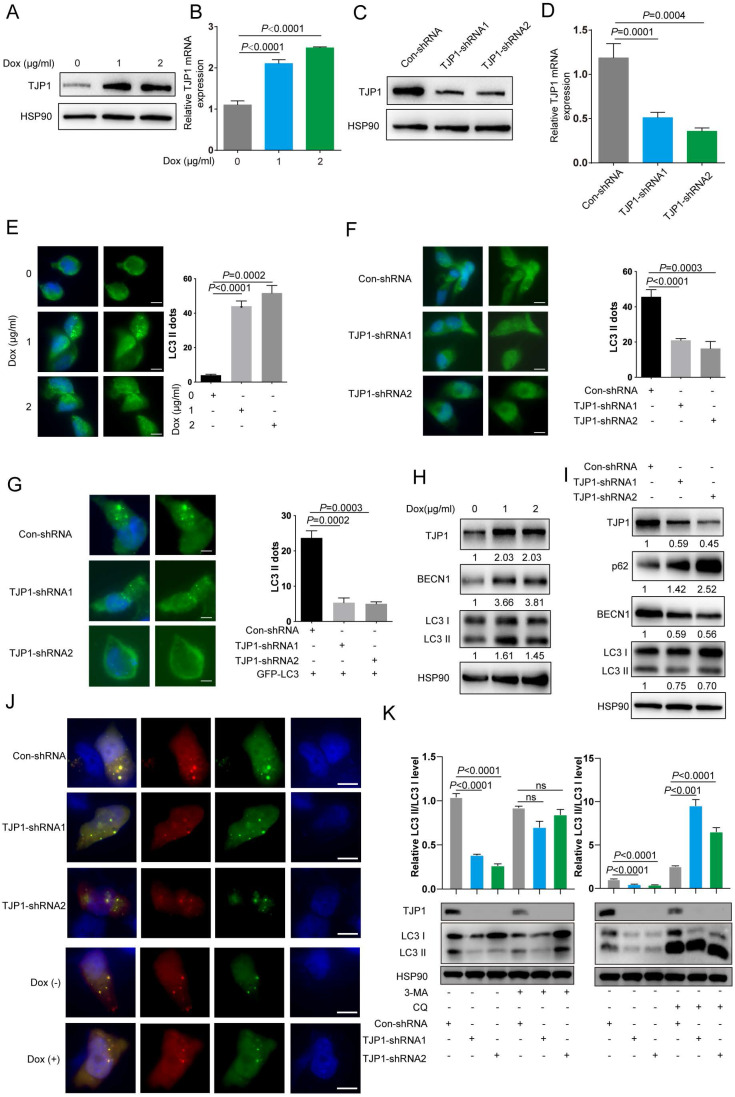
** TJP1 regulates autophagy signaling in 786-O cells. (A and B)** TJP1 protein (A) and mRNA (B) levels were analyzed in stable TJP1 inducible overexpressing 786-O cells (the indicated concentration of doxycycline induction for 24 hours) by western blotting and qRT-PCR respectively. **(C and D)** TJP1 protein (C) and mRNA (D) levels were analyzed in stable TJP1 knockdown 786-O cells by western blotting and qRT-PCR, respectively. **(E)** Representative images of immunofluorescence staining for aggregation of endogenous LC3 (anti-LC3B, green) and DAPI (blue) in the stable TJP1 inducible overexpressing 786-O cells (the indicated concentration of doxycycline induction for 24 hours). The scale bar = 10 μm. **(F)** Representative images of immunofluorescence staining for aggregation of endogenous LC3 (anti-LC3B, green) and DAPI (blue) in stable TJP1 knockdown 786-O cells. The scale bar = 10 μm. **(G)** Representative images of immunofluorescence staining for aggregation of LC3-GFP (green) and DAPI (blue) in stable TJP1 knockdown 786-O cells transfected with LC3-GFP plasmid. The scale bar = 10 μm. **(H)** LC3 II conversion and BECN1 protein levels were analyzed by western blotting in stable TJP1 inducible overexpressing 786-O cells (the indicated concentration of doxycycline induction for 24 hours). The gray-scale value was analyzed by ImageJ. **(I)** LC3 II conversion, BECN1 and p62 protein levels were analyzed by western blotting in stable TJP1 knockdown 786-O cells. The gray-scale value was analyzed by ImageJ. **(J)** The TJP1 kockdown or overexpression 786-O cells were transfected with LC3-GFP-RFP plasmids. Representative images of GFP- and RFP- dots were detected by fluorescence microscopy. Scale bar = 10 μm. **(K)** The protein level of LC3 I and LC3 II were analyzed by western blotting in stable TJP1 knockdown 786-O cells treated with 3-MA (left) or CQ (right). The relative intensity of LC3 II/LC3I was quantified with the ImageJ software and normalized with HSP90.

To examine the effect of TJP1 on autophagy, we constructed doxycycline-induced 786-O cells stably expressing TJP1 by using a lentivirus package system (786-O-Dox). When 786-O-Dox cells were treated with doxycycline at 1 or 2 μg/mL, the mRNA and protein level of TJP1 were significantly increased (Fig. 2A-B).

**Figure 3:**
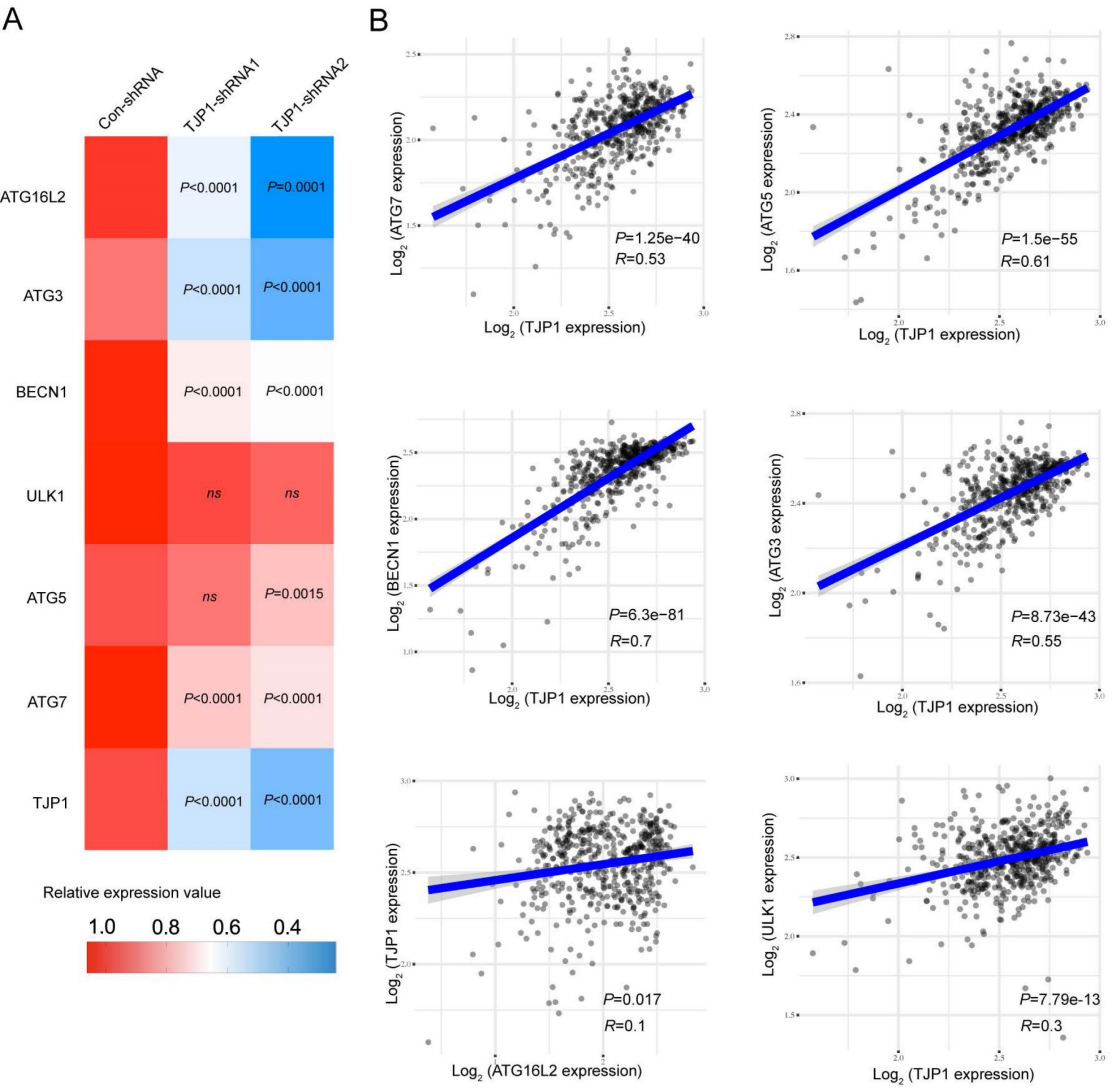
** TJP1 expression positively correlates with autophagy-related genes in KIRC. (A)** The mRNA expression of ATG16L2, ATG3, BECN1, ULK1, ATG5, ATG7, and TJP1 in stable TJP1 knockdown 786-O cells.** (B)** The correlation between TJP1 and ATG3, ATG5, ATG7, ULK1, BECN1, ATG16L2 expression were analyzed with Spearman in KIRC. The mRNA data was obtained from TCGA database.

We also constructed stable TJP1 knockdown 786-O cells by using two independent shRNAs targeting TJP1 (786-O-shRNAs). As shown in Fig. 2C and 2D, TJP1 expression was significantly knocked down at the protein and mRNA levels. We next visualized endogenous autophagic activity in 786-O-Dox cells by fluorescence microscopy. Compared to control cells, 786-O-Dox cells stably induced expressing TJP1 exhibited more LC3 puncta (Fig. 2E). Consistently, knockdown of TJP1 suppressed autophagy in 786-O cells (Fig. 2F). Another KIRC cell line OS-RC-2 exhibited the similar appearance (Fig. S1). Moreover, when exogenous LC3-GFP plasmids were transfected into 786-O-shRNA cells, LC3 fluorescence and LC3 puncta were markedly decreased in 786-O-shRNA cells (Fig. 2G). In addition, overexpression of TJP1 significantly promoted LC3 II conversion and upregulated BECN1, knockdown of TJP1 inhibited LC3 II conversion and p62 accumulation (Fig. 2H-I and Fig. S2). Autophagic flux consists of 3 sequential steps: autophagosome generation, autolysosome formation and degradation. We hypothesized that both autophagic flux and the formation of autophagosomes may have been the underlying cause of TJP1 overexpression-induced LC3 puncta generation. To determine whether TJP1 regulated autophagic flux in KIRC, we treated stable TJP1 knockdown 786-O cells with early phase and late phase autophagy inhibitors, 3-Methyladenine (3-MA) and chloroquine (CQ), respectively. We found that CQ but not 3-MA efficiently decreased LC3 II conversion in TJP1-knockdown cells (Fig. 2J and 2K). Collectively, these results indicate that knockdown of TJP1 suppresses the formation of autophagosomes but does not influence autophagic degradation, suggesting that TJP1 mainly regulates early steps during autophagy. As we detected p62 and BECN1 at the protein level, we further selected indispensable genes involved in these three steps. Surprisingly, we found that knockdown of TJP1 significantly inhibited ATG-related genes and BECN1 at the mRNA level, except ULK1 and ATG5 (Fig. 3A), whereas TJP1 expression had a positive correlation with these ATG-related genes in KIRC tissues (Fig. 3B). Taken together, these data suggest that TJP1 is an inducer of autophagy in KIRC cells.

### Autophagy-related genes correlates with favorable prognosis in KIRC

**Figure 4:**
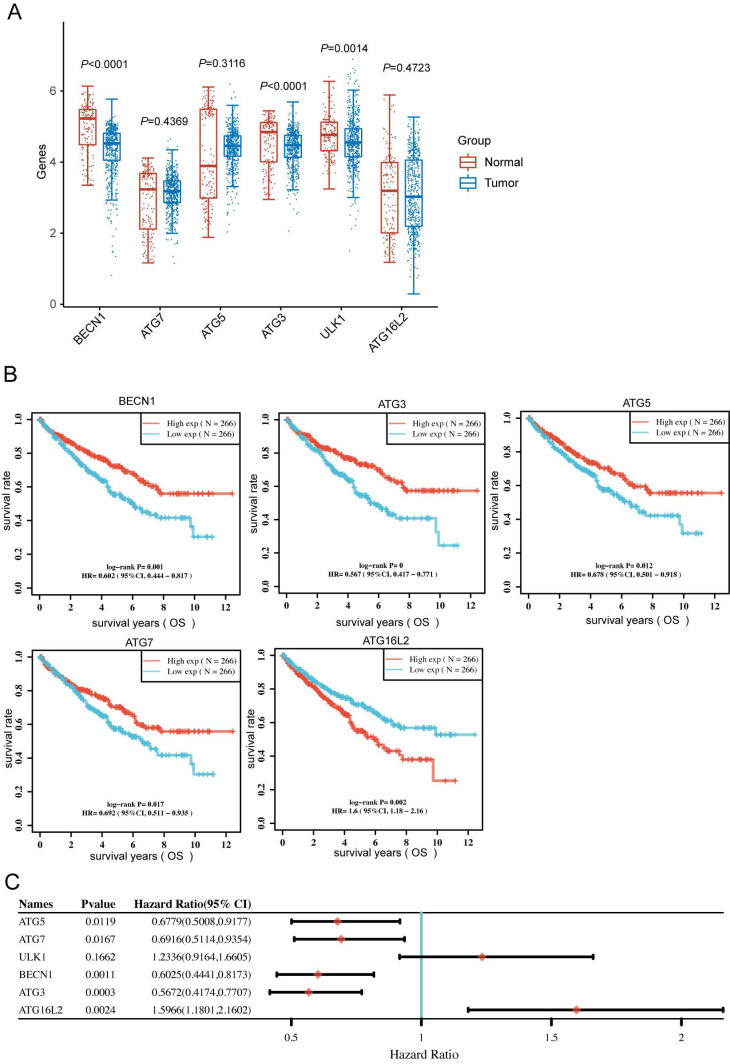
** Autophagy-related genes could predict prognosis of patients in KIRC. (A)** The BECN1, ATG7, ATG5, ATG3, ULK1 and ATG16L2 expression in primary KIRC tissues (n=533) and normal tissues (n=72). **(B)** Kaplan-Meier survival analysis of the patients based on gene expression (TPM: transcripts per million) of the indicated genes in KIRC. **(C)** Forest plot: The pvalue, risk coefficient (HR) and confidence interval of the indicated genes in KIRC are analyzed by univariate cox regression.

To clarify the BECN1, ATG7, ATG5, ATG3, ULK1 and ATG16L2 expression in KIRC patients, we downloaded RNA-sequencing expression profiles from the TCGA dataset. The expression of BECN1, ATG3, and ULK1 significantly altered between normal and tumor patients (Fig. 4A). Moreover, Kaplan-Meier survival analysis showed that high BECN1, ATG5, ATG7 and ATG3 expression significantly correlated with favorable prognosis, whereas high ULK1 and ATG16L2 expression correlated with poor prognosis (Fig. 4B and C). More importantly, we analysed the above autophagy-related genes with KIRC grade, which uncovered the significant expression of autophagy-related genes during tumor progression (Fig. S3).

### TJP1 inhibits KIRC cell proliferation in an autophagy-dependent manner

**Figure 5:**
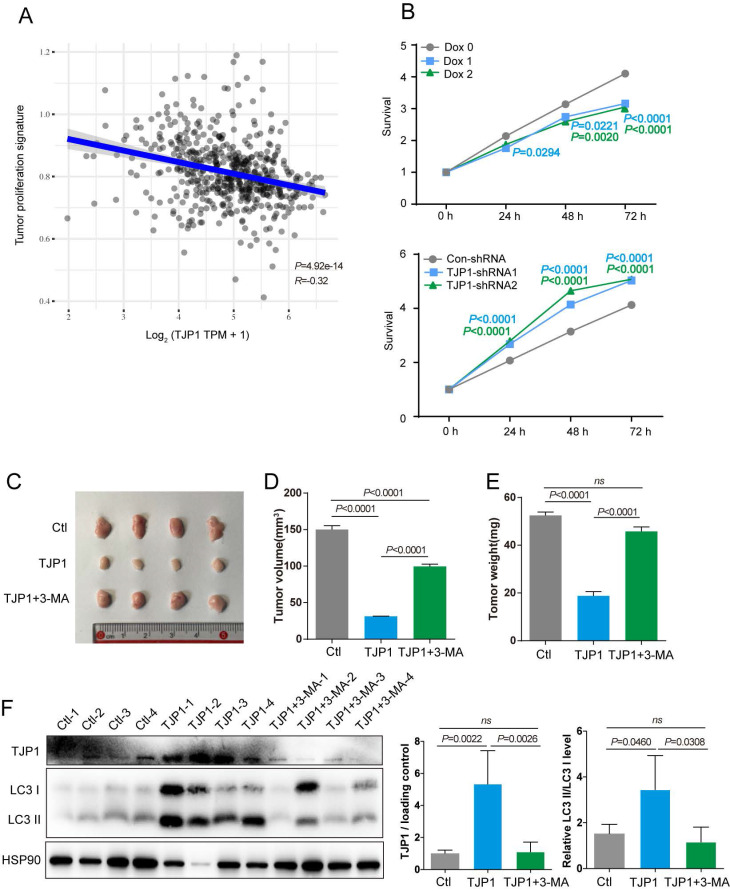
** TJP1 inhibits cell proliferation through promoting autophagy in KIRC cells. (A)** The correlations between TJP1 and tumor proliferation signature score was analysed with Spearman correlation. **(B)** The proliferation rate of stable TJP1 inducible overexpressing 786-O cells or stable TJP1 knockdown cells was analyzed by a CCK8 kit at the indicated time points. **(C-E)** The images of xenograft tumors (C), tumor volumes (D) and tumor weights (E) at endpoint from mice subcutaneously injected with 786-O cells stably expressing TJP1 treated with or without 3-MA, 786-O cells stably expressing vector as the control group. n = 4 biologically independent mice. **(F)** The protein levels of TJP1 and LC3 II conversion were analyzed by western blotting in the indicated xenograft tumors. The relative intensity of TJP1 and LC3 II conversion level were quantified with the ImageJ software and normalized with HSP90.

To determine function of TJP1 in KIRC, the correlations between TJP1 and the KIRC pathways were analyzed. We found that higher TJP1 expression was correlated with lower proliferation signature scores in KIRC (Fig. 5A). We next sought to confirm whether TJP1 could affect cell proliferation in KIRC cells. Knockdown of TJP1 significantly promoted cell proliferation in 786-O cells, and ectopic expression of TJP1 significantly suppressed cell proliferation in 786-O cells (Fig. 5B). Animal experiments were designed to detect the function of TJP1 *in vivo*. Mice injected with 786-O cells stably expressing TJP1 showed decreased tumor volume and weight compared to control group and mice treated with 3-MA (Fig. 5C-E). In addition, as shown in Fig. 4F, the tumors from mice injected with 786-O cells stably expressing TJP1 exhibited high LC3 II conversion along with TJP1 expression, whereas the tumors from 3-MA-treated mice exhibited lower LC3 II conversion level. All the above findings demonstrated that TJP1 overexpression could induce the autophagy to inhibit cell proliferation and tumor growth in KIRC cells.

## Discussion

TJP1 is one of the endothelial markers in the epithelial-mesenchymal transition (EMT) process and involved in tumor proliferation, cell communication, and angiogenesis, but the function of TJP1 in KIRC has not yet been explored. We first analyzed TJP1 expression in the TCGA database, considering clinical tumor stage and overall patient survival. The results demonstrated a correlation between decreased TJP1 expression, advanced tumor stage and poorer overall survival in KIRC (Fig. 1). To further elucidate the role of TJP1, we utilized 786-O cells with stable knockdown and overexpression of TJP1. Our experiments revealed that TJP1 inhibited cell proliferation and tumor growth by inducing the autophagy process. Dysregulation of TJP1 expression has been observed in various cancer types. Notably, in leiomyosarcoma, TJP1 knockdown led to the activation of multiple signaling pathways, including the NF-κB pathway and growth factor receptor signaling, resulting in enhanced tumor growth because of CSF1 and CTLA4 expression^26^. Similarly, in non-small cell lung cancer (NSCLC) cells, TJP1 was induced by TGF-β and contributed to the inhibition of cell motility^22^. Inflammatory bowel disease has also been associated with reduced TJP1 expression, as downregulation of TJP1 disrupts mucosal repair by attenuating Wnt-β-catenin signaling and inducing abortive epithelial proliferation 27, 28. Other tight junction proteins, such as TJP2, have been reported in colorectal cancer, hypopharyngeal squamous cell carcinoma and scirrhous gastric carcinoma^29^, ^30^.

Autophagy is a complex and dynamic physiological process that plays a critical role in tumorigenesis. It exhibits a dual role, with both pro-tumorigenic and anti-tumorigenic effects depending on the context, tumor type, and specific conditions. The interplay between autophagy and other signaling pathways in tumor cells is intricate and dynamic, influenced by the tumor microenvironment, genetic alterations, and external stimuli. Our results showed that TJP1 positively correlated with BECN1, ATG7 and ATG3 mRNA levels, which has not been reported in KIRC before (Fig. 3 and Fig. S3).

In mammals, the deletion of the BECN1-encoding region increases the risk of human breast, prostate, and ovarian cancer^31^-^33^. In human colon cancer, a single allelic mutated UV irradiation resistance-associated gene (UVRAG) is related to promoting autophagy and significantly inhibiting the proliferation of colon cancer cells^34^. EI24/PIG8 autophagy-related transmembrane proteins are also believed to promote tumor cell apoptosis and inhibit tumor function and have been reported to be mutated in breast cancer cells^35^. In addition to BECN1 and EI24, changes in ATG5 protein expression and somatic mutations of the ATG5 gene are also observed in gastrointestinal and prostate cancer^36^, ^37^. In our research, autophagy inhibitors confirmed that TJP1 mainly inhibited the early stage of the autophagy process, resulting in a decrease in LC3 II, which could be reversed by 3-MA (Fig. 2). In a xenograft model of KIRC, overexpressing TJP1 dramatically attenuated tumor proliferation, which could be restrained by 3-MA injection (Fig. 5). Relevant studies have shown that the autophagy-related PI3K/Akt/mTOR signaling pathway is closely related to disease progression. The combination of pathway inhibitors and autophagy inhibitors significantly inhibited the growth of 786-O cells and induced apoptosis. *Clostridium butyricum* protects intestinal barrier function via upregulation of claudin-1, claudin-2, occludin and TJP1, as well as the Akt/mTOR signaling pathway^38^. In the process of angiogenesis of glioblastoma, tight junction proteins and the PI3K/Akt/mTOR signaling pathway are consistently altered by celastrol^39^. mTOR is known to be a strong factor for inhibiting autophagy; mTORC1 phosphorylates ULK1 and suppresses its function, which in turn suppresses the formation of autophagosomes, and it is also the direct target of everolimus and temsirolimus clinical drugs. In colorectal cancer treatment, the combination of temsirolimus and chloroquine increases radiosensitivity^40^. In addition, temsirolimus can induce autophagy in adenoid cystic carcinoma by upregulating BECN1 and LC3 protein levels, leading to inhibition of tumorigenesis *in vivo* and *in vitro*^41^. Nevertheless, Ras/Raf/MEK/ERK signaling and AMPK signaling also alter cellular autophagy, particularly that of cancer cells. Meanwhile, TGF-β is an inducer of tight junction protein expression. TGF-β fucosylation enhances autophagy and mitophagy via PI3K/Akt and Ras/Raf/Mek/Erk in ovarian carcinoma^42^. Our analysis showed that high BECN1, ATG7 and ATG3 expression positively correlated with favorable prognosis, whereas low ULK1 and ATG16L2 expression negatively predicted the survival of KIRC patients (Fig. 4). Research reveals that ATG16L2 is indispensable in classical autophagosome formation, whereas ULK1 is the negative kinase in autophagy^43^, ^44^. Therefore, the above findings suggest that autophagy plays a crucial role in the favorable prognosis of KIRC, and that the activation of autophagy could serve as a beneficial treatment approach for patients.

Autophagy has been widely studied to regulate tight junction proteins. Our database analysis of KIRC revealed that TJP1 expression positively correlated with BECN1, ATG7 and ATG3. We also found that TJP1 knockdown decreased the BECN1, ATG7 and ATG3 transcriptional levels (Fig. 3). At the same time, other studies have found that hyperglycemia during early reperfusion after stroke increases the permeability of the blood-brain barrier and subsequently aggravates brain injury and clinical prognosis, mainly through MMP-2/9-mediated extracellular degradation and autonomic lysosome-mediated degradation of TJP1 protein^45^. Liao *et al*. found that exposure of human brain microvascular endothelial cells to the HIV protein Tat led to dose- and time-dependent induction of autophagy, with upregulation of BECN1, ATG5 and LC3B protein levels and consequent downregulation of TJP1, ultimately leading to increased cell permeability of endothelial cell monolayers in an *in vitro* blood-brain barrier model^46^. Liu *et al*. discovered that TJP1 can act as a mediator of mTOR to regulate the circadian rhythm of the liver by interacting with period circadian regulator 1 (PER1) and preventing its nuclear translocation^47^. In breast cancer, Song *et al*. revealed that the tight junction protein CLDN6 induces autophagy by positively regulating BECN1, thereby mediating the inhibitory effect of estrogen receptor β on breast cancer cell migration and invasion^48^. Studies have shown that UVRAG can act as a binding protein of BECN1 to mediate the activation of the BECN1-PI3KC3 complex to promote autophagy and inhibit the proliferation and tumorigenicity of colon cancer cells^49^. Nevertheless, Takahashi et al. suggested that UVRAG can bind to the SH3 domain^50^. We speculate that TJP1 may bind to UVRAG proteins through the SH3 domain to form a complex, which needed further investigation in our future research. Furthermore, in the tumor microenvironment, different subsets of T cells play a crucial part in tumor progression, and some research has implied tight junction in T cells. The CD8 T cells participated in chronic inflammatory and cancer^51^, the alterations of tight junction in CD8 T cells closely correlated to induction of blood brain barrier^52^. Th17 cells has been widely investigated in cancer and autoinflammation, whereas tight junction protein related to ashma previously^53^. The function of TJP1 in tumor cell and inflammatory cell communication will be a spot worth studying in the future.

## Conclusions

Autophagy has emerged as a promising target in the treatment of RCC. However, due to the complexity of the autophagy process and its dependence on various environmental factors, research on autophagy in the context of renal cell carcinoma remains limited. The findings of our study have several important implications. Firstly, it provides the first evidence of the involvement of TJP1 in regulating autophagy and its impact on tumor behavior in KIRC. Furthermore, the study highlights the potential of TJP1 and autophagy-related genes as favorabble prognostic biomarkers in KIRC. Overall, this study contributes to the growing body of knowledge on autophagy in renal cell carcinoma and underscores the significance of TJP1 in modulating autophagy and its potential as a prognostic biomarker. It has important clinical implications for the development of novel diagnostic and therapeutic approaches in the management of KIRC.

## Supplementary Material

Supplementary figures.

## Abbreviations

3-MA: 3-Methyladenine
Baf. A: Bafilomycin
EMT: epithelial-mesenchymal transition
KIRC: kidney renal clear cell carcinoma
KIRP: kidney papillary cell carcinoma
KICH: kidney chromophobe cell carcinoma
NSCLC: non-small cell lung cancer
RCC: Renal cell carcinoma
TCGA: The Cancer Genome Atlas
UVRAG: UV radiation resistance-associated gene
ZO-1: zonula occludens 1
