# Supplementary material for: Tight Junction Protein 1 Suppresses Kidney Renal Clear Cell Carcinoma Cells Proliferation by Inducing Autophagy
Source: Int J Med Sci. 2023 Sep 11;20(11):1448–59. doi: 10.7150/ijms.81065 (PMC10542186; doi:10.7150/ijms.81065)
Supplement: Supplementary file 1 — Supplementary figures. [file ijmsv20p1448s1.pdf]

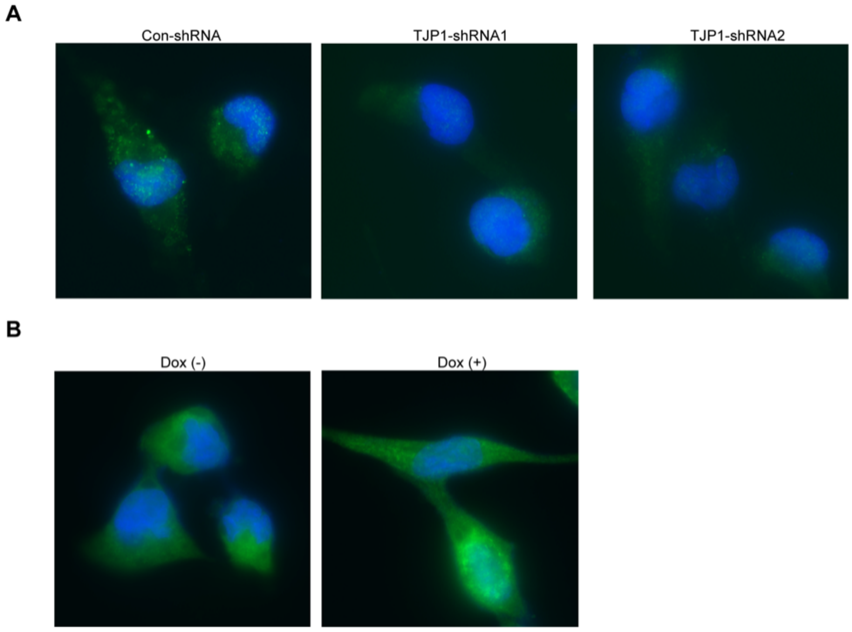

Fig S1. The LC3 fluorescence dots in OS-RC-2 cells.

(A-B) The LC3 fluorescence dots were detected in TJP1 knockdown (A) or overexpression (B) OS-RC-2 cells.

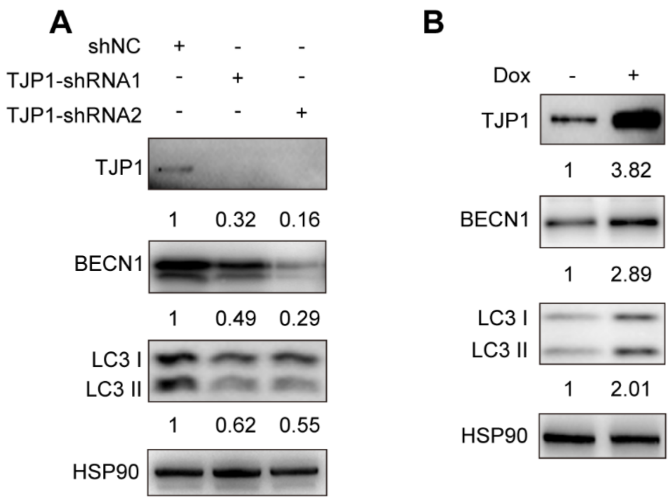

Fig S2. The analysis of autophagy in OS-RC-2 cell line.

(A-B) The TJP1 knockdown OS-RC-2 cells were constructed by lentivirus package plasmids and PLKO.1-shRNAs. The TJP1 overexpression OS-RC-2 cells were transfected with lentivirus package plasmids and Dox-inducible overexpression plasmid. The expression of TJP1, BECN1, LC3 and HSP90 protein were detected by western blotting in TJP1 knockdown (A) or overexpression (B) cell lines.

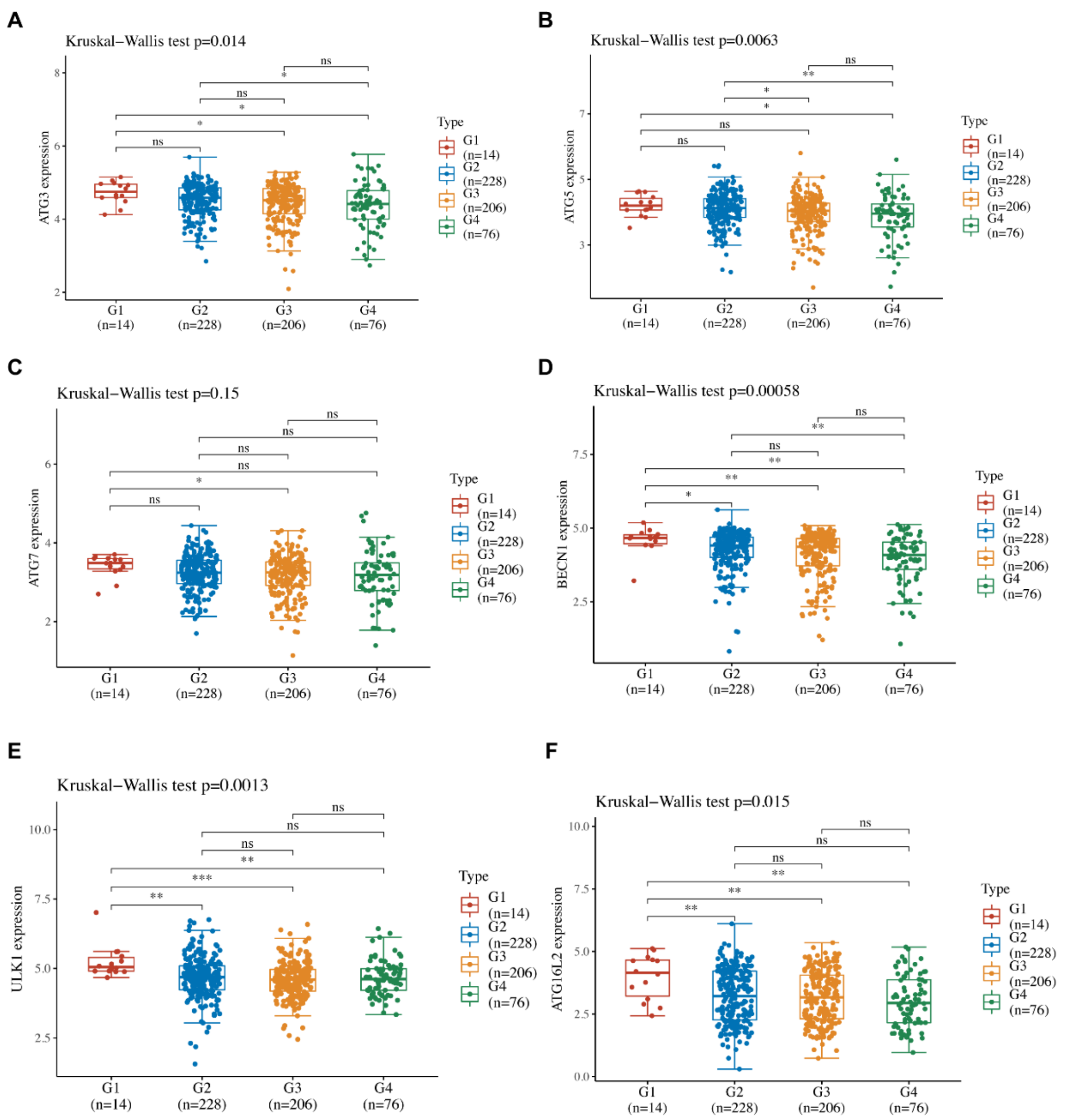

Fig S3. The analysis of autophagy and KIRC grade.

(A-F) The expression distribution of ATG3 (A), ATG5 (B), ATG7 (C), BECN1 (D), ULK1 (E) and ATG16L2 (F) gene in KIRC tumor tissues and normal tissues. The abscissa represents different grades of samples, and the ordinate represents the expression distribution of the gene, different colors represent different groups, top-left represents the significance *p*-value. *$p<0.05$, **$p<0.01$, ***$p<0.001$. The statistical difference of two groups was compared through the Wilcox test, significance difference of three groups was tested with Kruskal-Wallis test.
